# Mechanisms Underlying Overactive Bladder and Interstitial Cystitis/Painful Bladder Syndrome

**DOI:** 10.3389/fnins.2018.00931

**Published:** 2018-12-12

**Authors:** Luke Grundy, Ashlee Caldwell, Stuart M. Brierley

**Affiliations:** ^1^Visceral Pain Research Group, Centre for Neuroscience, College of Medicine and Public Health, Flinders University, Adelaide, SA, Australia; ^2^Centre for Nutrition and Gastrointestinal Diseases, Discipline of Medicine, South Australian Health and Medical Research Institute, The University of Adelaide, Adelaide, SA, Australia

**Keywords:** bladder, overactive bladder, interstitial cystitis, afferent, peripheral, central, sensitisation

## Abstract

The bladder is innervated by extrinsic afferents that project into the dorsal horn of the spinal cord, providing sensory input to the micturition centers within the central nervous system. Under normal conditions, the continuous activation of these neurons during bladder distension goes mostly unnoticed. However, for patients with chronic urological disorders such as overactive bladder syndrome (OAB) and interstitial cystitis/painful bladder syndrome (IC/PBS), exaggerated bladder sensation and altered bladder function are common debilitating symptoms. Whilst considered to be separate pathological entities, there is now significant clinical and pre-clinical evidence that both OAB and IC/PBS are related to structural, synaptic, or intrinsic changes in the complex signaling pathways that mediate bladder sensation. This review discusses how urothelial dysfunction, bladder permeability, inflammation, and cross-organ sensitisation between visceral organs can regulate this neuroplasticity. Furthermore, we discuss how the emotional affective component of pain processing, involving dysregulation of the HPA axis and maladaptation to stress, anxiety and depression, can exacerbate aberrant bladder sensation and urological dysfunction. This review reveals the complex nature of urological disorders, highlighting numerous interconnected mechanisms in their pathogenesis. To find appropriate therapeutic treatments for these disorders, it is first essential to understand the mechanisms responsible, incorporating research from every level of the sensory pathway, from bladder to brain.

## Introduction

Overactive bladder syndrome (OAB) and interstitial cystitis/painful bladder syndrome (IC/PBS) are common, chronic, pelvic disorders affecting approximately ∼16% of the western population (Hanno, 2002; Irwin et al., 2006; McLennan, 2014; Truzzi et al., 2016). Urgency, frequency, and nocturia are common symptoms of both OAB and IC. However, these conditions may be differentiated by the presence of urge urinary incontinence in patients with OAB and pelvic pain in IC patients (MacDiarmid and Sand, 2007; Hanno and Dmochowski, 2009; Haylen et al., 2010; Homma et al., 2016). As both of these disorders are diagnosed in the absence of bacterial infection or obvious pathology, the etiology of OAB and IC/PBS symptoms remain unknown. Accordingly, efficacious therapeutic options are limited, contributing to the significant societal and economic impact of greater than $70 billion per annum in the United States (Pierce and Christianson, 2015; Durden et al., 2018).

**FIGURE 1:**
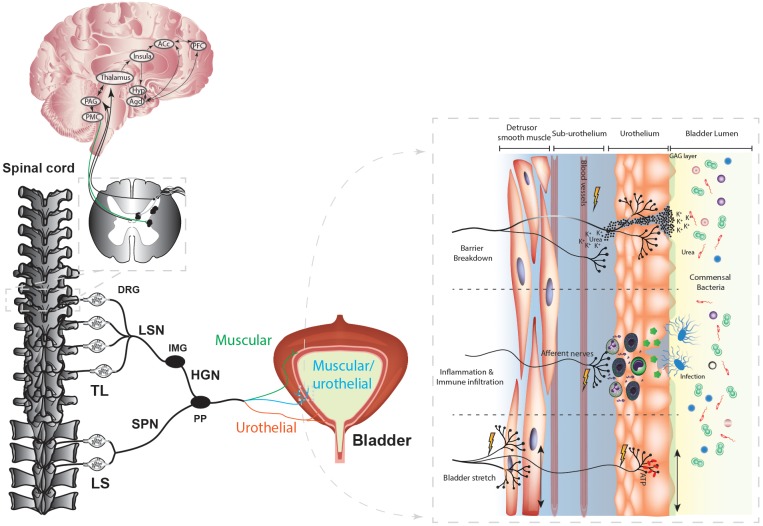
Spinal innervation of the bladder. The afferent nerves innervating the bladder wall extend into the detrusor smooth muscle to detect bladder stretch, and into the bladder urothelium to detect bladder stretch, infection, inflammation, and barrier breakdown. The bladder lumen contains numerous commensal bacteria as well as toxic contents such as urea and high potassium. Bacterial infection of the urothelium induces apoptosis of urothelial cells, the release of cytokines, and the infiltration and activation of the immune response, including mast cell degranulation and the subsequent release of histamine and cytokines that can sensitize bladder afferent neurons. During bladder stretch the urothelium releases an array of neurotransmitters, including ATP, which can activate bladder afferents. Breakdown of the urothelial barrier allows access of toxic urine contents into the underlying bladder interstitium which can activate and sensitize bladder afferents. Bladder afferents project via the pelvic nerve or the splanchnic nerve to the dorsal horn of the thoracolumbar and lumbosacral spinal cord, where they activate second order neurons within the spinal cord synapse in the thalamus or the PAG of the midbrain. Thalamic projections provide input into limbic and cortical structures to provide the emotional affective and conscious component of the voiding reflex pathway. The thalamus relays to the PAG and the PAG feeds into the PMC to signal micturition. TL, Thoracolumbar; LS, lumbosacral; SC, spinal cord; PFC, Prefrontal Cortex; ACc, Anterior cingulate cortex; Hyp, hypothalamus; HGN, hypogastric nerve; PAG, periaqueductal gray; PMC, primary micturition center; DRG, dorsal root ganglion; DH, Dorsal horn; Agd, amygdala; LSN, lumbar splanchnic nerve; SPN, pelvic nerve; IMG, inferior mesenteric ganglion; PP, Pelvic Plexus. Figure modified from Grundy et al. (2018c).

Normal bladder function requires coordination of afferent signals originating from the bladder wall with excitatory and inhibitory signals from the anterior cingulate cortex (ACC), insula, and hypothalamus to provide an overview of the appropriateness to urinate that is ultimately under conscious control by the prefrontal cortex (Figure 1; Griffiths, 2015; Lovick, 2016). Bladder afferents embedded within the detrusor smooth muscle show exquisite sensitivity for mechanical distension but are also found innervating the urothelium (Zagorodnyuk et al., 2006, 2007, 2009; Spencer et al., 2018). This topology provides a secondary level of resolution to the transmission of sensory stimuli, including the detection of bladder infection, urothelial inflammation, or barrier breakdown (Figure 1). Accordingly, bladder sensory afferents express a range of anti- and pro-nociceptive receptors and ion channels (Erickson et al., 2018; Grundy et al., 2018c) that integrate the input from this complex signaling environment and can induce a range of sensations from fullness through to pain (Fowler et al., 2008). These afferents, whose cell bodies are located within the dorsal root ganglia (DRG), project via the pelvic, hypogastric/splanchnic nerves, synapse within the dorsal horn of the lumbosacral (LS, L5-S1) and thoracolumbar (TL, T10-L2) spinal cord (Figure 1; Fowler et al., 2008; de Groat and Yoshimura, 2015) and terminate within the periaqueductal gray (PAG) (Fowler et al., 2008). The PAG acts as an integration center for afferent signals from the spinal cord and higher brain centers (Griffiths, 2015). A conscious “urge” to urinate is perceived when afferent activity increases beyond a pre-set threshold and, if modulating input from the brain permits, the PAG activates the pontine micturition centre (PMC) to induce efficient voiding (Andersson and Arner, 2004; Fowler et al., 2008; Griffiths, 2015; Lovick, 2016).

Clinical studies reveal that patients with OAB and IC/PBS perceive sensations of bladder fullness, urge to void, and pain at lower cystometric volumes than healthy subjects (Van Brummen et al., 2004; Kim et al., 2009; Parsons and Drake, 2011). These observations thereby implicate the sensitisation of bladder afferent pathways to physiological stimuli as a key component in the mechanisms underlying these disorders (Yamaguchi et al., 2007; Yoshimura et al., 2014; de Groat and Yoshimura, 2015). Furthermore, co-morbidity of depression and anxiety is significantly higher in patients with OAB and IC/PBS, while high psychological stress levels is strongly correlated to exacerbated bladder symptoms (Goldstein et al., 2008; Lai et al., 2015; Golabek et al., 2016; Leue et al., 2017; McKernan et al., 2017). As such, increased intensity of the afferent signal or modulation of the emotional affective state can have profound effects on bladder sensation. A key concept therefore, in the development of OAB and IC/PBS, is chronic sensitisation of the neuronal networks regulating bladder sensation, incorporating structural, synaptic, or intrinsic changes of peripheral or central structures that may drive subsequent changes in downstream sensory pathways (Brierley and Linden, 2014; Grundy et al., 2018c).

In this review, for brevity, we summarize both preclinical and clinical research to highlight how alterations in peripheral afferent excitability contribute to the symptoms of OAB and IC/PBS to provide insights into the mechanisms that are hypothesized to mediate these distinct disorders which have many overlapping symptoms. For an excellent review on the potential changes in central processes occurring in OAB see Reynolds et al. (2016).

## Urothelial Permeability

Toxic waste metabolites excreted in urine are prevented from accessing the bladder interstitium and embedded afferent endings by a tight urothelial barrier (Spencer et al., 2018; Figure 1). The urothelial barrier is maintained by tight junctions between apical urothelial cells, hydrophobic uroplakin plaques, and a considerable glycosaminoglycan (GAG) mucus layer, that block the movement of small molecules and urine (Birder and Andersson, 2013; Hurst et al., 2015). From a clinical point of view, numerous studies have identified that patients with IC/PBS, but not OAB, have a diminished or damaged urothelium (Elbadawi and Light, 1996; Tomaszewski et al., 2001; Lai et al., 2013; Keay et al., 2014; Hurst et al., 2015), as well as reduced expression of the tight junction proteins zona occludens 1 (ZO-1) and E-cadherin (Liu et al., 2012). Increased bladder permeability due to reduced urothelial integrity is thought to underlie the sensitivity of IC/PBS patients (75%) to the potassium sensitivity test (PST) compared to control patients (4%) (Parsons et al., 1998). A number of clinical studies also show that OAB patients respond to the PST, however, this may be due to overlap and misdiagnosis between IC/PBS and OAB (Minaglia et al., 2005; MacDiarmid and Sand, 2007; Chung et al., 2010). Despite these observations, it remains to be determined if bladder permeability is part of the underlying pathology of bladder hypersensitivity in IC/PBS patients or a downstream consequence of localized inflammation that further exacerbates the condition.

Glycosaminoglycan replacement therapy with pentosane polysulfate (PPS) has been shown to improve the symptoms for some, but not all, IC/PBS patients (Nickel et al., 2012; Lai et al., 2013; Oliveira et al., 2014; Hurst et al., 2015). However, PPS also induces broad anti-inflammatory actions, including the inhibition of mast cell histamine release in the bladder (Chiang et al., 2000; Anderson and Perry, 2006; Wu et al., 2011; Sanden et al., 2017). In addition, pre-clinical studies inducing urothelial permeability with protamine sulfate identified a relatively rapid recovery of the urothelial barrier and structure (Lavelle et al., 2002; Greenwood-Van Meerveld et al., 2015), via injury-induced proliferation of basal urothelial and stromal cells (Shin et al., 2011). A more likely scenario therefore is that bladder permeability in IC/PBS patients is secondary to localized inflammation. Continuous access of toxic urine contents combined with inflammatory mediators, such as cytokines, histamine, and proteases, can sensitize peripheral afferent endings and have the potential to trigger long term changes in neuronal function and neuroplasticity within the entire afferent network (Brierley and Linden, 2014; Grundy et al., 2018c).

### Altered Release of Urothelial Factors

In addition to its role as a physical barrier, the urothelium provides bi-directional communication with underlying primary afferents (Lazzeri, 2006; Birder and Andersson, 2013; Merrill et al., 2016) via the detection and/or release of a range of excitatory and inhibitory neurotransmitters and neuromodulators including ATP, acetylcholine, nitric oxide (NO), NGF, prostaglandin E2 (PGE2), neurokinin A, and inflammatory mediators as described above (and extensively reviewed by Birder et al.) (Everaerts et al., 2010a,b; Birder and Andersson, 2013; de Groat and Yoshimura, 2015; Grundy et al., 2018a). Altered urothelial mediator release has been identified from OAB and IC/PBS patients in a number of studies and may be a compounding mechanism in the development of chronic neuronal hypersensitivity (Kim et al., 2005, 2006; Sun and Chai, 2006; Suh et al., 2017).

Chronic bladder inflammation enhances ATP release from the urothelium and augments purinergic signaling of bladder afferents in rats (Smith et al., 2005), whilst both stretch-mediated ATP release and its receptor P2X_3_ are increased in the urothelium of OAB and IC/PBS patients (Sun and Chai, 2004, 2006; Contreras-Sanz et al., 2016; Jhang and Kuo, 2016). Pre-clinically, acetylcholine, acting via muscarinic receptors also triggers release of ATP, as well as NO and prostanoids from urothelial cells (Winder et al., 2014; Michel, 2015). In animal studies, experimentally induced cystitis upregulates muscarinic receptors and acetylcholine release, as well as subsequent cholinergic regulated urothelial NO release (Giglio et al., 2005; Giglio and Tobin, 2009; Andersson, 2011; McDermott et al., 2013). Notably, PGE2 levels are significantly increased in OAB patients (Kim et al., 2005, 2006), whilst NO and PGE2 levels and receptor expression are also altered in IC/PBS patients with Hunner’s legions (Wada et al., 2015; Jhang et al., 2016). Bradykinin stimulates urothelial NGF release and enhances stretch-induced ATP release in a human urothelial cell line (Ochodnicky et al., 2013; Winder et al., 2014), whilst bradykinin 1 (B1) receptor expression is upregulated in human IC/PBS patient bladder samples (Arms and Vizzard, 2011) and in the urothelium of a rat CYP-induced cystitis model (Chopra et al., 2005).

Limited causations have been determined for altered urothelial neurotransmitter release or receptor expression in OAB and IC/PBS, but there is accumulating evidence that they are a downstream consequence of inflammation, infection or urothelial breakdown.

## Inflammation

By definition, the presence of bladder inflammation precludes the clinical diagnosis of OAB. Only a small population of IC/PBS patients exhibit significant inflammation, which is characterized by the presence of Hunner’s ulcers (Leiby et al., 2007). However, it is widely reported that there are increases in the amount of pro-inflammatory mediators within the bladder and urine of both OAB and IC/PBS patients (Kastrup et al., 1983; El-Mansoury et al., 1994; Hauser et al., 2008; Jacobs et al., 2010; Liu et al., 2012; Jhang and Kuo, 2016; Furuta et al., 2018). These pro-inflammatory mediators including histamine, nerve growth factor (NGF), and those released from mast cells, are known to directly sensitize afferent nerve terminals (Davidson et al., 2014). Furthermore, overexpression of pro-inflammatory genes, oedema, tissue granulation and an increase in macrophages, chemokines, cytokines, eosinophils, as well as T and B cell markers have also been identified in IC/PBS patients (Hauser et al., 2008; Abernethy et al., 2017). As the majority of cystitis-induced inflammation is localized to the superficial mucosa, urothelial afferents are ideally placed to detect and respond to these environmental changes.

Inflammation-induced sensitisation of afferents is an essential mechanism for the induction of normal wound healing, however, chronic sensitisation of afferents can occur during prolonged inflammation or following a severe bout of inflammation (Brierley and Linden, 2014; Abraham and Miao, 2015). In support of an inflammatory-mediated pathophysiology in urological disorders, animal models of cystitis have employed a range of chemicals, including acetic acid, acrolein, cyclophosphamide (CYP), zymosan and lipopolysaccharide, that induce both acute and longer lasting bladder hyperactivity (Takezawa et al., 2014; Liu and Dong, 2015; Abdi et al., 2016; Hughes et al., 2016). Whilst there are many limitations to the use of inflammatory animal models in the study of symptom defined disorders such as OAB and IC/PBS (comprehensively discussed by Fry et al., 2010), these animals show an overactive bladder phenotype, with altered cystometry and enhanced visceromotor response during bladder distension, replicating the reduced bladder capacity, plus the allodynia and hyperalgesia to bladder distension observed in humans (Fry et al., 2010; Lai et al., 2011; DeBerry et al., 2014, 2015b). Furthermore, bladder afferents show direct sensitisation to chemical and inflammatory stimuli (de Groat and Yoshimura, 2009), whilst retrogradely traced bladder-innervating DRG neurons from CYP-treated rats or a naturally occurring feline interstitial cystitis model exhibit lower activation thresholds and sensitisation to current injection (Dang et al., 2008; Buffington, 2011). The efficacy of intraluminal therapies to treat OAB and IC/PBS (Cvach and Rosamilia, 2015; Manriquez et al., 2015), that either (1) block bladder afferent firing (such as lidocaine and neosaxitoxin) or (2) cause peripheral nerve desensitization (with agents such as resiniferatoxin; RTX), highlight the important role of peripheral afferents in mediating bladder hypersensitivity to distension (Apostolidis et al., 2005).

The transient receptor potential (TRP) channel TRPV1 is upregulated in the bladders of patients with OAB and IC/PBS (Liu and Kuo, 2007), whilst both TRPV1 and TRPA1 have consistently been implicated in mediating normal and cystitis-induced mechanical sensitivity in rodents by modulating neuronal activation thresholds and enhancing bladder afferent responses to P2X receptor activation (Daly et al., 2007; Wang et al., 2008; DeBerry et al., 2014, 2015b; Yoshiyama et al., 2015; Grundy et al., 2018b). A host of additional receptors and channels associated with nociception have also been identified upon bladder afferents that regulate neuronal sensitivity and neuronal excitability in animal models of cystitis, including voltage gated sodium (Na_V_) channels (Erickson et al., 2018; Grundy et al., 2018d), potassium channels (K_V_) (Hayashi et al., 2009), P2X receptors (Dang et al., 2008; Chen and Gebhart, 2010), TRPV4 (Merrill et al., 2012) and cannabinoid receptors (Hedlund, 2014; Izzo et al., 2015; Bakali et al., 2016; Hedlund and Gratzke, 2016; Munoz, 2016).

It is possible that a population of patients present without active inflammation or increased bladder permeability but are in fact in remission from a preceding bladder infection or inflammation. Such a scenario could induce a protracted hypersensitive state and correspond to their enhanced sensory symptoms. Indeed, women with a clinical history of recurrent UTI as children are significantly more likely to have a diagnosis of IC/PBS as adults (Peters et al., 2009), and preclinical investigations of neonatal bladder insult in rats suggests this may be due to long term sensitisation of sensory pathways (Randich et al., 2006; DeBerry et al., 2007; Ness and Randich, 2010). Neuroplasticity of peripheral afferent circuitry following the resolution of inflammation or recovery from tissue injury has been well documented in both somatic and visceral pain models through the induction of neurogenic inflammation and neuronal sprouting (de Groat and Yoshimura, 2009; Gregory et al., 2013; Brierley and Linden, 2014). Neonatal bladder inflammation in rats results in hypersensitive responses to inflammatory stimuli as an adult, inducing an overactive bladder phenotype (Randich et al., 2006; DeBerry et al., 2007, 2010), as well as enhanced spontaneous and urinary bladder distension-evoked activity of spinal visceral nociceptive neurons (Ness and Randich, 2010). Alterations in spinal cord circuits responsible for bladder sensation may regulate this phenomenon, as neonatal inflammation induces a downregulation of GABA (Aα-1) receptor microRNA and altered opioid peptide content in the dorsal horn (Sengupta et al., 2013; Shaffer et al., 2013). Furthermore, neonatal zymosan enhances bladder neuropeptide content of CGRP and Substance P compared to sham controls (DeBerry et al., 2010; Shaffer et al., 2011).

Similarly, in pre-clinical models of adult cystitis, bladder overactivity is associated with increases in tyrosine receptor kinase (Trk) A, Trk B, and calcitonin gene-related peptide (CGRP) (Vizzard, 2001; Qiao and Vizzard, 2002), which in turn promote inflammation in the tissue where the afferent terminals reside (Rosa and Fantozzi, 2013). Furthermore, patients with IC/PBS have higher elevated serum and urinary NGF levels than healthy controls (Chen et al., 2016). NGF overexpression in mouse urothelium leads to neuronal hyper-innervation, increased mast cell counts and changes in bladder function (Schnegelsberg et al., 2010). These discoveries may explain the increased sprouting of neuronal terminals identified in the bladders of IC/PBS patients (Christmas et al., 1990; Lundeberg et al., 1993), that has been replicated in rodent models of inflammation *in vivo* and *in vitro* (Dupont et al., 2001; Schnegelsberg et al., 2010; Boudes et al., 2013; Ekman et al., 2017).

## Microbiome/Chronic Urinary Tract Infection

Following the recent identification of a bladder-specific microbiome, and the role of the gut microbiome in chronic functional gastrointestinal diseases (Hughes et al., 2013; Harper et al., 2018), a link between the balance of bacteria in the bladder and the symptoms of OAB and IC/PBS has been postulated and explored (Contreras-Sanz et al., 2016; Angelini, 2017; Drake et al., 2017). Moreover, the traditional colony forming unit thresholds for confirming urinary tract infection (UTI) in clinical practice have been questioned, and a role for chronic UTI in the pathogenesis of OAB has been investigated (Balachandran et al., 2016).

Patients with OAB may have genuine uropathogenic infections, and are therefore misdiagnosed, as large numbers of bacteria are undetected by routine mid-stream urine cultures (Khasriya et al., 2013). Indeed, a significantly greater number of patients with refractory idiopathic detrusor overactivity show low count bacteriuria vs. controls (Walsh et al., 2011). Undiagnosed intracellular bacterial colonization of urothelial cells may also occur in OAB (Scott et al., 2015), as OAB patients exhibit significantly greater infected urothelial cell counts and microscopic pyuria than healthy subjects, which also correlates to urgency symptoms (Gill et al., 2018). Uropathic *E. coli* infection initiates the release of multiple mediators from the urothelium, including cytokines and interleukins, as well as promoting urothelial barrier defects (Wood et al., 2012), which alert the immune system to impending damage and initiate an immune response (Abraham and Miao, 2015). Immune cell infiltration and the release of pro-inflammatory cytokines are known to sensitize peripheral afferents (Ren and Dubner, 2010), and in this way enhance bladder sensation. In support of these considerations, a recent pilot study revealed that combination antibiotic treatment of both Gram-negative and Gram-positive bacteria significantly improved OAB symptoms as well as the perception of their bladder condition (Vijaya et al., 2013). Furthermore, shifts in the bacterial species that constitute the bladder microbiome have been associated with both the presence and severity of OAB and IC/PBS (Siddiqui et al., 2012; Whiteside et al., 2015; Contreras-Sanz et al., 2016; Lakeman and Roovers, 2016; Curtiss et al., 2017). For example, women with IC/PBS, but not OAB, have a less diverse microbiota than those without (Hilt et al., 2014; Pearce et al., 2014; Abernethy et al., 2017; Curtiss et al., 2017). Interestingly, despite significant inter-patient variability in bladder microbiome, a decrease in *Lactobacillus*, which has antimicrobial properties, in both OAB and IC/PBS patients compared to controls is a common finding (Hilt et al., 2014; Pearce et al., 2014; Curtiss et al., 2017). Furthermore, the absence of *Lactobacillus acidophilus* correlates with higher pain scores and higher scores on the interstitial cystitis symptom index (Abernethy et al., 2017). *Proteus*, the urinary pathogen, is also identified more commonly in patients with OAB and lower urinary tract symptoms than healthy controls (Khasriya et al., 2013; Curtiss et al., 2017). These data support a line of communication between the urinary microenvironment and underlying afferent nerves that is likely mediated by the urothelium.

### Cross-Organ Sensitisation

Considerable clinical evidence suggests that diseases of the colon, such as irritable bowel syndrome (IBS) and inflammatory bowel disease (IBD), can induce subsequent development of pathology in an otherwise unaffected adjacent organ, such as the bladder (Grundy et al., 2018d).

A mouse model of colitis induced by intra-rectal instillation of 2,4,6-trinitrobenzene sulfonic acid (TNBS) induces hyper-excitability of the entire peripheral sensory pathway, from the afferent ending in the colon to the spinal cord (Brierley and Linden, 2014). Importantly, TNBS colitis also prompts consistent changes in bladder voiding parameters that replicate the clinical symptoms of urgency and frequency, as well as increased bladder-afferent sensitivity to bladder distention (Brumovsky and Gebhart, 2010; Ustinova et al., 2010; Greenwood-Van Meerveld et al., 2015; Yoshikawa et al., 2015). These symptoms occur in the absence of any overt inflammation or histological damage to the bladder, highlighting the importance of altered afferent sensitivity in maintaining OAB and IC/PBS symptomology (Yoshikawa et al., 2015).

This “cross-organ sensitisation” is considered to originate within the physiological co-ordination of these pelvic organs, and persistent pathological plasticity of their shared sensory pathways within the thoracolumbar and lumbosacral DRG and spinal cord (Persson et al., 2015; Grundy et al., 2018d). Approximately 15% of colonic innervating TL and LS DRG neurons exhibit dichotomising afferents, simultaneously innervating the bladder (Christianson et al., 2007; Yoshikawa et al., 2015), whilst a similar proportion of spinal dorsal horn neurons also respond to both urinary bladder and colonic distension (Grundy et al., 2018d). As such, sensitisation of colonic afferent pathways has the potential to directly influence the excitability of bladder afferent pathways. Indeed, a very recent pre-clinical study also indicates that chronic sensitisation of colonic afferent pathways results in the subsequent sensitisation of bladder afferent pathways and the triggering of uncontrolled urinary voiding in mice. Intriguingly, these changes in bladder function can be reversed by a therapeutic treatment targeted only to the colon (Grundy et al., 2018e).

Additionally, neurochemical changes occur within colonic afferent pathways following colitis that indicate the development of neurogenic inflammation (Grundy et al., 2018d). Multiple studies have shown that this translates to persistent upregulation of the neuromodulators NGF, BDNF, CGRP, and the high affinity receptor TrkB in bladder, bladder-innervating DRG neurons, and spinal cord (Liang et al., 2007; Qiao and Grider, 2007; Pan et al., 2010; Xia et al., 2015; Kawamorita et al., 2016). These receptors and neuropeptides share an intimate relationship enabling the positive feedback of each other (Malykhina et al., 2006; Christianson et al., 2007; Lei et al., 2013; Yoshikawa et al., 2015), consequently inducing neuronal sensitisation and neurite outgrowth, and likely contributing to cross-organ sensitisation through paracrine actions within the ganglia to increase bladder afferent excitability (Xia et al., 2015; Sorkin et al., 2018).

### Cortical Regulation: Stress, Anxiety, and Depression

The sensory signals from bladder afferent converge in the PAG where they are modulated by input from the limbic system (amygdala, hypothalamus, thalamus, cingulate gyrus), insula, and prefrontal cortex (Fowler et al., 2008), which can in turn modulate or be modulated by the hypothalamic pituitary adrenal (HPA) axis. As such, changes to cortical networks or modulation of the emotional affective state can have profound effects on bladder sensation and may be an underlying mechanism in the development and persistence of OAB and IC/PBS symptoms.

Patients with OAB and IC/PBS report psychological stress levels that are significantly higher than healthy controls (Lai et al., 2015), which may be a consequence of HPA axis dysregulation following chronic early life stress (ELS) (Taylor, 2010). Exposure of children to ELS is a significant risk factor for developing HPA abnormalities (Anand, 1998; Pierce and Christianson, 2015), and shows strong correlations with the development of depression and anxiety in later life (Egeland, 2009; Heim and Binder, 2012). To this end, a number of studies report higher incidences of ELS and trauma in IC/PBS patients than healthy controls (Fuentes and Christianson, 2018), whilst clinical studies have demonstrated a strong correlation between stress, anxiety, depression, and the symptoms of bladder overactivity in patients with OAB and IC/PBS (Goldstein et al., 2008; Golabek et al., 2016; Leue et al., 2017). The increased prevalence of depression and anxiety in patients with IC/PBS occurs both following and prior to bladder symptoms, indicating a reciprocity in cause and effect, with no clear way to delineate patient cohorts to provide increased mechanistic understanding (McKernan et al., 2017). In addition to relationships with cognitive disorders, structural abnormalities within the white matter of the brain in women with IC/PBS, which facilitates the communication between and within brain regions, correlates closely to symptom severity (Farmer et al., 2015). It remains unclear whether these white matter properties are causes or consequences of IC/PBS. It is possible that certain white matter architecture may reflect a predisposition to develop disease, but it is equally plausible that these changes are a consequence of IC/PBS disease progression and future longitudinal studies are required to test this hypothesis.

The link between stress and bladder disorders is supported by pre-clinical studies in rodents that consistently induce bladder overactivity or mechanical hyperalgesia following stress-treatments (Black et al., 2009; Merrill et al., 2013; DeBerry et al., 2015a; Lee et al., 2015; Wang et al., 2017). In addition, this hypersensitivity has been found to be dependent on (DeBerry et al., 2015a), or correlate with, significant changes in brain regions associated with emotional processing and bladder control (Wang et al., 2017). Neonatal maternal separation in female mice, as a model of ELS, enhances visceromotor responses to urinary bladder distension accompanied by altered hippocampal input onto the HPA axis (Pierce et al., 2016). If sensitisation of peripheral afferent endings occurs in these models as a consequence of stress, or if sensitisation is arbitrated solely within the CNS has yet to be fully determined.

## Conclusion

This review highlights the complex nature of both OAB and IC/PBS, incorporating evidence for changes in the dynamic signaling environment between the bladder lumen, urothelium, and afferent nerves, coordinating with adaptations to the HPA axis, and emotional affective components of sensory processing mediated within the limbic system. The bladder microbiome, bacterial infection, inflammation, and urothelial permeability contribute to the development of peripheral afferent hyperexcitability that is fundamental to the development of frequency and urgency in OAB, and pain in IC/PBS. In addition, the higher psychological stress levels, increased prevalence of anxiety and depression, as well as clinical co-morbidities with other visceral pain disorders suggests pathological plasticity within the CNS is an important component in the mechanisms underlying both OAB and IC/PBS. Determining the underlying mechanisms of bladder hypersensitivity is paramount to providing novel targets for the development of safer and more efficacious treatments.

## Author Contributions

AC and LG performed literature searches. AC, LG, and SB wrote the manuscript. All authors made significant contributions to the formation and correction of the manuscript in preparation for submission.

## Conflict of Interest Statement

The authors declare that the research was conducted in the absence of any commercial or financial relationships that could be construed as a potential conflict of interest.
